# Sound and Video Detection as a Tool to Estimate Free Grazing Behavior in Sheep on Different Swards

**DOI:** 10.3390/ani15182671

**Published:** 2025-09-12

**Authors:** Marcella Avondo, Matteo Bognanno, Francesco Beritelli, Roberta Avanzato, Luisa Biondi, Filippo Gimmillaro, Salvatore Bognanno, Alessandra Piccitto, Serena Tumino

**Affiliations:** 1 Dipartimento di Agricoltura, Alimentazione e Ambiente, University of Catania, 95123 Catania, Italy; luisa.biondi@unict.it (L.B.); filippo.gimmillaro@unict.it (F.G.); salvatorebgn@gmail.com (S.B.); alessandra.piccitto@unict.it (A.P.); serena.tumino@unict.it (S.T.); 2 Dipartimento di Scienze e Tecnologie Agro-Forestali e Ambientali, University of Reggio Calabria, 89100 Reggio Calabria, Italy; matteo.bognanno@unirc.it; 3 Dipartimento di Ingegneria Elettrica, Elettronica e Informatica, University of Catania, 95123 Catania, Italy; francesco.beritelli@unict.it (F.B.); roberta.avanzato@unict.it (R.A.)

**Keywords:** grazing sounds, sheep, free grazing behavior, legumes sward, grass sward, point of view camera

## Abstract

The aims of the study were to evaluate the effectiveness of audio detection for identifying feeding sounds in free grazing sheep and to assess whether the recognition of these sounds could be influenced by pasture characteristics. Twelve Valle del Belice dry ewes were grazed on two mixed swards: on 10 May, grass-rich sward (G); on 13 May, legume-rich sward (L). Each ewe was fitted with a collar equipped with a point of view (POV) camera. All audio files (without viewing the videos) were listened to and sounds recognized as herbage prehension and rumination activity were highlighted. Time spent eating and ruminating was then calculated. To validate the audio file analysis, all video files were subjected to observation of the same behavioral aspects detected with audio. The regression between the prehensions number estimated using sound alone and the actual values recorded through video was significant (r^2^ 0.743; *p* < 0.001). No differences were found in recognizing grazing behavior between data obtained by listening or watching the videos and between the two swards. The acoustic analysis of the single bites on grass and legume forages reveals significant differences between the two forage classes (*p* ≤ 0.001) particularly in terms of energy, temporal structure, and spectral features. Since sheep showed a strong selective activity towards legumes even in the grass-rich sward (selectivity index 3.1), this may have reduced acoustic differences between swards.

## Simple Summary

Precision Livestock Farming (PLF) technologies enable the monitoring and acquisition of data relating to animal activities across various stages of farming. In a sheep farming system, the study of grazing sounds using recorders has allowed the clear recognition of activities such as herbage prehensions and rumination, thus characterizing grazing behavior. By employing point of view (POV) cameras, it was possible to recognize the aforementioned sounds and individual prehensions in two complex pastures (a grass-rich and a legume-rich swards). Different sound features have been highlighted between grass and legume prehensions. Significant linear regression for the number of herbage prehensions, eating and ruminating time was found between the values estimated solely from sound recordings and the actual values monitored via video analysis. Recording the number of bites is a key element for estimating herbage intake in grazing animals. Knowledge of this information provides valuable insights for improving both animal and pasture management, with positive consequences for production and environmental sustainability, even in extensive farming systems.

## 1. Introduction

Understanding the grazing behavior of ruminants can have a positive impact on productivity, products quality and metabolic well-being. Due to the difficulty of collecting behavioral data through direct observation, which involves following animals at short distances and recording their activities during grazing, alternative detection systems to direct observation have been developed. Among the various systems, the detection of the head position by means of triaxial accelerometers is probably the most frequently adopted method by researchers. Herbage prehension, chewing, and rumination activities or resting can be recognized using this technique [1,2], although the data are not always reliable due to the incorrect classification of head movements as prehensions [3]. This can lead to overestimation of prehension and rumination movements [4].

Video and audio recording represents another available tool for detecting feeding behavior. Sound studies, mostly conducted on cattle, have often been carried out under extremely controlled conditions, for example, with micro-swards fed to animals for short periods, in conditions of almost no external sounds [4,5,6,7]. Several sound classification systems have been developed with good performances [6,8,9,10,11]. The adaptation of the above classification systems to the ovine species could represent a possible alternative to the creation of new tools. Nevertheless, under less controlled grazing conditions, as occurs in actual sheep farming practice, loud background noises, such as the sound of bells, the wind, or the noise of nearby animals, can make the detection of feeding behavior through audio recording more challenging than in the controlled situations adopted in the abovementioned studies. Jin et al. (2022) [12] emphasize that automatic classification systems designed for cattle should not be adapted to sheep due to behavioral and physiological differences between species.

Few researchers focused on sheep feeding behavior by using sounds. Milone et al. (2009) [6] in highly controlled grazing conditions, using micro swards, showed good performance of an automatic method of recognition of chewing events in sheep. Galli et al. (2020) [4], in similar controlled conditions, found that accuracy in classifying jaw movements was better for cows than sheep. Wang et al. (2021) [13] found a model that classifies foraging behavior in sheep with an accuracy of 93%. However, the classification was based on a very limited dataset, and the experimental conditions were very uncomfortable for the animals. Indeed, sheep had a collar connected to a rope so that the experienced staff affected sheep’s natural movement during grazing activity.

To the best of our knowledge, only one study available in the literature [14] has been conducted with sheep fed in real grazing conditions and not on artificial swards. These authors found that automatic recognition of jaw movements was more accurate in cattle and goats than in sheep, where the false positive rate 24% compared to 7 and 4%, respectively. In fact, when sheep graze, sound is often severely disturbed by noises resulting from a different ingestion behavior compared to cattle.

In most studies on the modeling of sounds detected during grazing, preliminary recognition of grasping, chewing, ruminating, and other activities was achieved through video recordings with standard digital cameras. Therefore, to film the animal’s mouth, it was necessary to follow it, risking disturbing its natural feeding behavior. The alternative often used is to film the animals with fixed cameras positioned in front of the animal, but these involve very controlled grazing situations, on specially created artificial swards. The use of point of view (POV) cameras overcomes several limitation of traditional video recording, such as poor lighting conditions, distance from the animals, and the potential disturbance caused by following them.

Animal-borne cameras have been widely used to study the behavior of wild animals [15] and domestic cats [16]. To our knowledge, only a few studies tested the use of these cameras to detect grazing sheep behavior, during daylight hours [17] and during the night [18]. The same authors considered this approach the only valid system for detecting grazing animal behavior, demonstrating that POV cameras provide a tool for highlighting the microstructure of the bites in animals grazing on complex grasslands rich in diverse botanical species. For this reason, we believe that the use of POV cameras represents the most effective system to characterize with certainty the feeding behavior of grazing animals

The aim of the study was to evaluate, using POV cameras, the effectiveness of audio detection in recognizing typical feeding sounds in free-ranging sheep. The study also aimed to assess whether the recognition of these sounds could be influenced by pasture characteristics.

## 2. Materials and Methods

All of the animals were managed according to the guidelines of the Animal Ethics Committee (O.P.B.A.) of the University of Catania (prot. No.232046).

The study was conducted using 12 dry Valle del Belice ewes. All sheep grazed on two swards of 0.5 Ha with different botanical composition according to the following plan: 10 May, mixed sward with a predominance of grass (G); 13 May, mixed sward with a predominance of legumes (L) (Table 1).

The temperature during grazing hours on 10 and 13 May was 24 and 25 °C, respectively; there was no precipitation and the wind speed ranged between 7.5 and 8.0 km/h on both days.

### 2.1. Biomass, Chemical and Botanical Composition of Swards

At the beginning of the two grazing days, three 0.5 × 0.5 m squares randomly distributed throughout the grazed areas, were cut at the base of each plant. The cut herbage was weighed, and each species was separated, classified into three classes (grasses, legumes and other essences) and weighed. All samples of the individual species from each 0.5 × 0.5 m square were subjected to near-infrared reflectance spectroscopy according to ISO 12099:2017 [19]. The chemical composition of each plot was then calculated using weighted averages.

### 2.2. Grazing Behavior Detection

**Figure 1:**
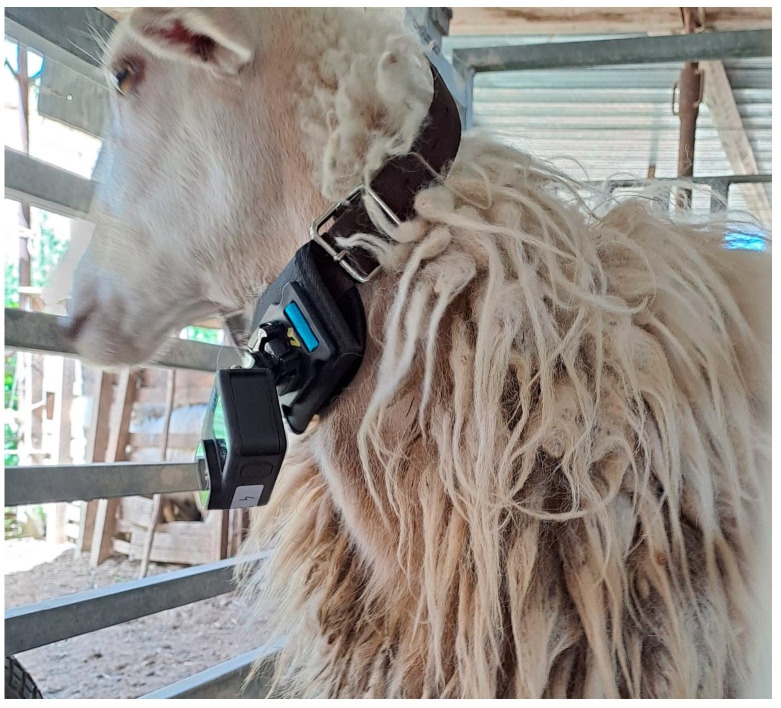
An experimental ewe fitted with a POV camera attached to collar.

All ewes were fitted with a collar equipped with a POV camera (GOPRO HERO 11, GoPro, Inc., San Mateo, CA, USA) (Figure 1).

The animals were left to graze for about 90 min per day. Each video was automatically segmented by the POV camera into 7–8 videos of approximately 12 min. Each of these short videos was analyzed separately and, therefore, for statistical purposes, it was considered as an experimental unit. A total of approximately 36,000 herbage prehensions were classified.

Selective behavior was assessed for each video, by noting the selected species every 60 s. At the end of all the observations, the percentage of grasses, legumes, and other essences was calculated. The selectivity index (SI) for the different forage species, calculated as the ratio between the percentage of selected species and the percentage of the same species present in the pasture, was evaluated according to the classification proposed by Stuth (1991) [20]: SI < 0.7, rejected; SI 0.7–1.3, neutral; SI 1.3–2.5, preferred; SI > 2.5, highly preferred.

### 2.3. Audio File Analysis

**Figure 2:**
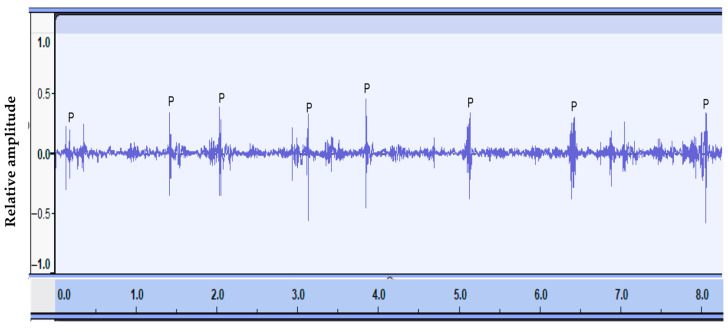
Waveform of audio signals related to herbage prehension. Each prehension is indicated by the letter P.

In the first phase, all audio files produced by the cameras (without viewing the videos) were listened to by specially trained researchers. By listening to the sounds in WAV format and viewing the graphic representation of the sound wave, sounds recognized as herbage prehension (Figure 2) and rumination activity were highlighted using a dedicated web application, named BioAcoustic Labeler (BAL), specifically developed by our research team at the University of Catania. The tool was designed to visualize the audio signal, annotate prehension and rumination events, and listen to specific segments in loop. The BioAcoustic Labeler produces CSV files with timestamps indicating the start and end of each activity, which were then used to calculate, for each audio file, the time spent eating, ruminating, and performing other activities during the observation period.

**Figure 3:**
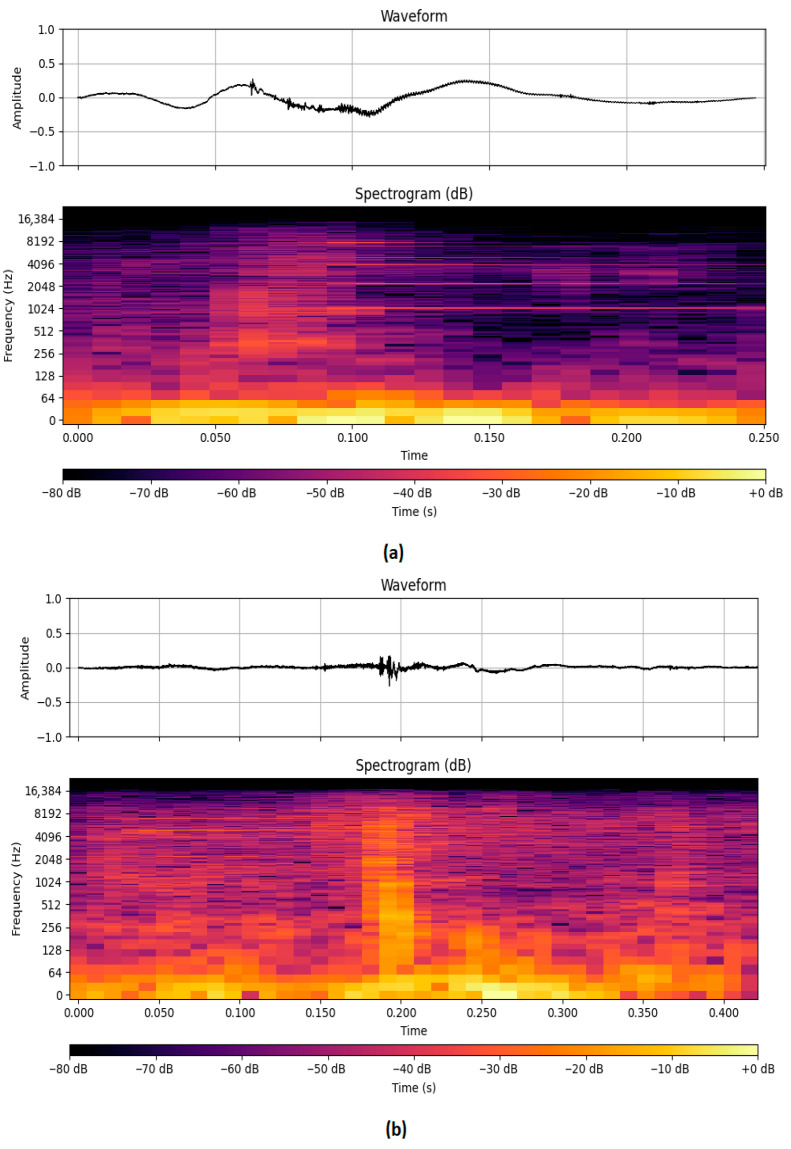
Time and Frequency Domain relating to audio segment. (**a**) Grass and (**b**) legume prehension.

To evaluate if bites on legumes and grass differ in terms of sound characteristics, 60 audio segments of grass and 50 of legumes were randomly selected by observing the corresponding videos. For each audio segment, a set of temporal and spectral acoustic features was extracted to characterize the prehension events. The Duration of each segment was computed from the annotated start and end timestamps, providing an estimate of the bite length. The Power, in decibel Full Scale (dBFS), offered a logarithmic measure of that energy, independent of normalization. The Pitch, representing the perceived fundamental frequency, was estimated to capture harmonic patterns related to the biting action. The Zero Crossing Rate (ZCR) was used to quantify the number of times the signal crosses zero, serving as an indicator of high-frequency content or signal discontinuity. Each segment was also converted into its frequency domain representation using a spectrogram on a logarithmic scale, allowing for visual comparison of spectral content between classes with a consistent color range from –80 dB to 0 dB (Figure 3).

### 2.4. Video File Analysis

**Figure 4:**
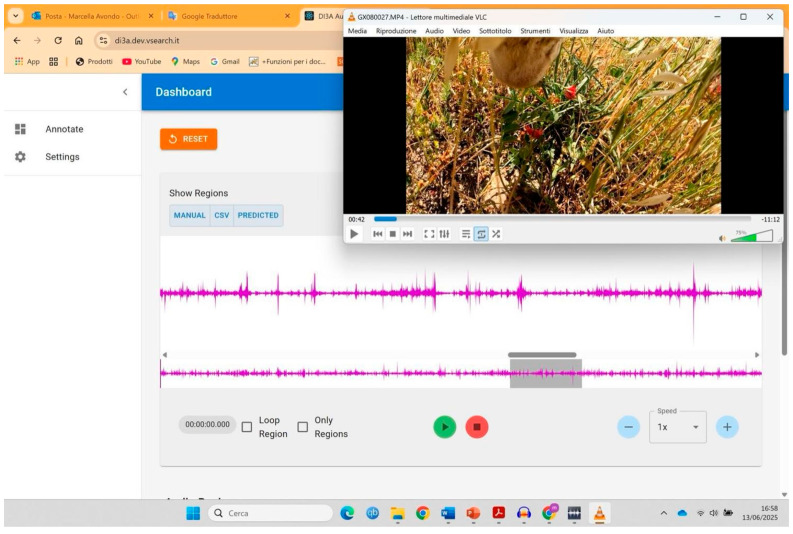
Example of juxtaposition and synchronization of audio and corresponding video files.

Following audio analysis, all video files were subjected to observation. The aim was to validate the audio file analysis through visual recording of the same behavioral aspects detected with audio recordings alone: number of herbage prehensions and rumination activity, or other activities. Each observed event was highlighted using the same tool used for the audio, juxtaposing and synchronizing each audio file with the corresponding video (Figure 4). This allowed us to identify true positive, false positives and false negatives.

The botanical composition of the selected diet was determined by video observation: for each video the choice of botanical species was observed every 1 min. Therefore, for each ewe, this choice was observed approximately 90 times.

### 2.5. Calculation of Time of Ingestion, Rumination and Other Activities

The estimated (audio) and actual (video) ingestion time were calculated from the CSV files generated by listening to the audio and watching the videos, according to the following criterion: ingestion is characterized by phases dedicated to prehension and rapid, coarse chewing of the bite, interspersed with phases in which the sheep is stationary or moves in search of other areas to graze. The ingestion time was therefore calculated by adding the time elapsed, for each ingestion phase, from the first to the last prehension, followed by an interval of more than 10 s. This interval was chosen arbitrarily based on the observations made considering that, in our experimental conditions, the rapid chewing of the bite just ingested does not last beyond this interval. Therefore, it can be considered that for more than 10 s, the animal is stationary or moving in search of other pasture areas. All intervals between prehensions exceeding 10 s were considered “other activities.” The time spent ruminating was calculated by adding the rumination periods (from bolus regurgitation and chewing to swallowing), excluding inactivity exceeding 10 s.

### 2.6. Statistical Analysis

The SPSS 26 package was used for statistical analysis. Data relating to ingestion time, rumination time, time spent on other activities, and number of prehensions were subjected to an univariate general linear model, considering the detection method (audio only; audio supported by video) and the type of pasture (grasses; legumes) as fixed factors, as well as their interaction. Correctly detected prehensions (true positives), false positives, false negatives, chemical and botanical composition of the pasture, selected essences, and selectivity indices were subjected to an univariate general linear model, considering the type of pasture (grasses; legumes) as a fixed factor. Duration, Power, Pitch, and Zero Crossing Rate (ZCR) data were analyzed by an univariate general linear model, considering the type of plants (grass or legumes) as a fixed factor.

All data detected by listening to the audio files relating to ingestion and rumination time and number of prehensions were subjected to simple linear regression analysis, using the respective data collected by observing the video files as independent variables.

To explore the distribution of the extracted audio features, descriptive statistical analysis of prehension classes (grass and legumes) was performed using boxplots. This approach provides a clear visual representation of each feature across classes, highlighting differences in central tendency, variability, and the presence of potential outliers.

## 3. Results

### 3.1. Swards Characteristics

Table 1 shows the biomass, chemical, and botanical composition of the mixed swards. Sugar and fat content were significantly higher in the grass-rich sward. Regarding the chemical composition of single botanical species, in both swards, grass was lower in crude protein and higher in sugars and NDF than legumes (on average: crude protein, 10.6 vs. 16.4% dry matter (DM); sugars, 15.2 vs. 11.4% DM; NDF, 63.5 vs. 35.8% DM). The other species had crude protein and sugar contents very similar to grass but lower NDF levels (on average: crude protein, 10.7% DM; sugars, 13.9% DM; NDF, 45.5% DM). The botanical composition of the swards and the selected diets and their corresponding selectivity indices (SI) are shown in Table 2. Among the botanical species, despite the evident differences in floristic composition between the two swards, only legumes percentage was significantly influenced by the sward type.

The percentage of legumes in the selected diet, as expected, was significantly higher in the sward with a prevalence of legumes. However, the selectivity index for legumes was significantly higher in the sward with a prevalence of grasses. The percentage of other essences in the selected diet was significantly higher in the sward with a prevalence of grasses.

### 3.2. Grazing Behavior

On average, the grazing behavior measurements, time spent eating, ruminating or doing other activities and the number of prehension (Table 3), did not show significant differences between swards, except for the time spent on other activities, which was significantly greater in the legumes-rich sward. No differences were found between the measurements conducted by listening and by watching the videos. The interaction between the main factors was never significant. Percentages of true positives, false positives and false negatives, precision and recall, obtained by the comparison between herbage prehensions estimated with audio recordings and detected with video recordings in the two swards (Table 4) were not significantly affected by the sward type.

Linear regression between data collected with sound alone and with sound combined with video was significant (*p* value > 0.001) for the number of prehensions, the eating time and the rumination time in both pastures (Table 5). However, a very high R^2^ was found for rumination in grass-rich sward compared to a much lower, though still highly significant, R^2^ in legume-rich sward.

### 3.3. Audio Characteristics of Grass and Legumes Prehensions

**Figure 5:**
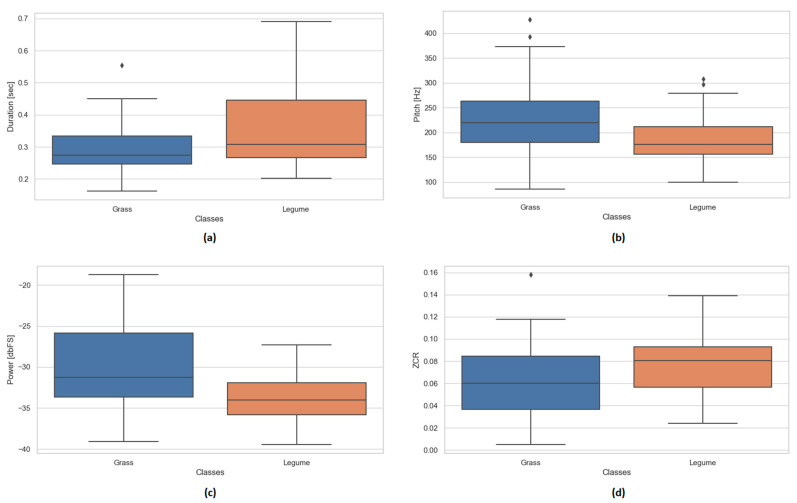
Boxplots of the main acoustic features extracted from audio segments for the two classes: grass- and legume bites. (**a**) Duration, (**b**) Pitch, (**c**) Power [dbFS], (**d**) Zero Crossing Rate (ZCR).

For each of the main acoustic features arising from the signals registered by ewes biting grass or legume plants, boxplots were computed to compare the distributions between the grass and legume bites sounds. Figure 5a shows that the duration of the bite segments is generally longer and more variable for the legume forage, while the grass forage tends to produce shorter prehensions with harder consistency. The pitch values are higher on average in the grass forage (Figure 5b), with a broader distribution and some extreme values, whereas the legume forage presents a more compact range centered around lower frequencies. Figure 5c illustrates the power in dBFS, indicating that the sound produced by ewes eating grass tends to have higher acoustic energy (closer to 0 dBFS), while legume bites are associated with lower power levels. Lastly, Figure 5d compares the zero crossing rate (ZCR), where the legume forage shows slightly higher median values, suggesting a richer high-frequency content or more discontinuous signals. The audio characteristics (Table 6) were significantly different between plant types: grass bites showed lower duration and ZCR and higher pitch and power, compared to legume bites.

## 4. Discussion

The subjective framing of POV cameras clearly highlighted all the animals’ behavioral activities without any disturbance that could interfere with their normal behavior. Therefore, in our experimental conditions, the sounds detected in the presence of POV camera videos, clearly identified and classified as prehension, rumination, or other activity, represented real data, while the sounds classified in the absence of videos were considered estimated data. No animals showed behavioral signs of discomfort due to the collar-mounted cameras.

In the majority of studies on grazing animal sounds, the recorders were mounted on the forehead [4,6,13]. These recorder positions captured sounds produced by the skull following the various mouth activities. This highlighted sounds that were classified as bite, chew, and chew-bite, the latter resulting from chewing during grasping. In our conditions, since the sounds were acquired from the collars, events such as chew-bites or chewing alone, often very recognizable but at other times much less so, were not recognized. This was because, in our case, the vibration of the skull was missing. However, in our opinion, the advantage of recording sounds from a collar allows for the acquisition of a clearer sound of individual herbage bite, independent of chewing activity. These bites, in fact, represent the most important variable for herbage intake estimation; indeed, herbage intake is calculated by the product of eating time x number of bites x bite mass [21].

Sheep are grazers with a high capacity for selection, especially when using pastures characterized by high biodiversity in terms of botanical composition [22], and therefore, they often penetrate the vegetation in search of the most palatable plants or plant parts. Furthermore, as gregarious animals, they tend to graze very close together, so the noises produced by neighboring animals can make challenging the recognition of feeding sounds.

In our experimental conditions, the noises associated with the selective activity for preferred species produced strong rustling sounds, which nevertheless did not prevent the recognition of prehensions. Furthermore, even the grasping noises of adjacent ewes did not prevent us from recognizing the herbage prehensions. The weather conditions during the 2 days of recordings, characterized by a lack of rain and a light wind, did not interfere with the acquisition of grazing sounds. These conditions were particularly favorable. However we believe that even the presence of wind should not pose any problems in detecting prehension sounds, having had other experiences with cattle grazing [11] in the presence of wind reaching up to 48 km/h. Despite the very loud noise recorded, the sound of the herbage prehensions was still audible, which stands out for its peculiar characteristics even against loud background noise. This is because the grasping sound is recorded at a very short distance from the animal’s mouth.

The R^2^ values obtained from the regression analysis between the values estimated using sound alone and the actual values monitored through video, together with the absence of significant differences between mean values acquired with audio and video, demonstrate that listening the sounds obtained through a recorder placed in the animal’s collar provides a reliable estimate of eating and rumination time, as well as the number of prehensions.

The second objective of the study was to evaluate whether the different types of pasture used on the two days of observation (grass sward vs. legume sward) could influence the recognition of grazing activity events (prehension, rumination, other activities). This hypothesis was formulated during the sound labeling phase; in fact, the noise of tearing grass generally seemed more intense and different from the sound perceived when the sheep ingested legumes or other essences. Observing the video together with the sound allowed us to note that when the sheep chose grass, the bite consisted of a group of stems which are characterized by a higher level of fiber than legumes (NDF value: 63.5 vs. 35.8% DM, respectively, in grass and legumes forages), whereas when the sheep chose legumes, the grasps were mostly directed at individual stems or leaves. This resulted in a rapid succession of sounds due to the severing of individual stems within each grass bite. Legume bite, on the other hand, consisted of the severing of an individual leaf or stem. The hypothesis was therefore that prehensions of grasses could be easier to recognize, compared to legumes.

The two swards were very different from each other in terms of both available biomass and botanical composition, with consequent differences in chemical composition as well. However, statistical significance of the differences between pastures was achieved only for a few parameters (percentage of legume forages, WSC, and fat). The lack of statistical significance for all other chemical-nutritional parameters of the two pastures is probably due to the high variability found between the different samples collected within each pasture.

The acoustic analysis of sheep prehension events on grass and legume forages, based on ANOVA results and on boxplot inspection of key audio features, reveals systematic differences between the two classes of sward. These differences pertain to temporal, spectral, and energy-related aspects of the recorded bite events. Segment duration emerged as a key differentiating factor. Legume bites were longer and more variable. In contrast, the grass bites showed more consistent, shorter events, indicating quicker and more stereotyped prehensions. Pitch analysis showed higher fundamental frequencies in the grass class, possibly due to more abrupt or forceful mandibular activity, while the legume class exhibited lower and more stable pitch values. This could reflect a smoother, less percussive prehension process when dealing with softer legumes. Power (dBFS) also showed clear separability between bite classes. The grass class exhibited both higher average power and greater variability, potentially due to more vigorous chewing or denser plant structure. Notably, while the current analysis was conducted on sheep, previous studies in cattle reported partially contrasting observations. For instance, Galli et al. (2020) [4], although not detecting significant differences between grasses and legumes, noted that chewing sounds associated with legumes tended to exhibit greater energy and shorter duration. Li et al. (2021) [23] found that tall fescue produced bite sounds with greater amplitude and duration compared to alfalfa. Our results, which indicate higher energy and shorter durations for grass forage prehensions, align only partially with these trends, suggesting that species-specific chewing strategies, forage texture, and bite mechanics may play a crucial role in shaping the acoustic profiles. These discrepancies highlight the importance of species-specific investigations and reinforce the need for dedicated acoustic datasets in sheep. Despite the observed differences in the acoustic characteristics between grass and legume prehensions, no significant differences were found between the two swards in the recognition of the different grazing sounds such as, eating, rumination, and other activities. This may be partially explained by the intense selective activity towards legumes also observed in the sward with a prevalence of grasses. Grass was the least selected (rejected according to the classification proposed by Stuth (1991) [20]) in both swards, despite the high percentage present in the grass-rich one. Legumes were highly preferred in the grass-rich sward where despite the minimal percentages present in the pasture (3.4%), the selection reached 10.1%, corresponding to a selective index of 2.96. The other species were preferred in both forage fields, compared to the available percentages.

Based on these results, it cannot be stated with certainty that the presence of grasses can improve the recognition of herbage prehensions, as initially hypothesized. Statistical analysis showed no effect of pasture type on ingestion and rumination times or on the number of prehensions.

## 5. Conclusions

POV cameras allowed for a deep analysis of sheep grazing behavior in real grazing conditions, allowing for the precise identification of herbage prehensions and diet selection activity on two different swards. Sound detection from a collar allowed for high-probability recognition of the number of prehensions, the time spent eating, and the time spent ruminating in both swards, thus demonstrating the effectiveness of this method in detecting typical feeding activity in free-ranging sheep. The results confirm the existence of significant differences in the acoustic signals of bites between the two types of forage analyzed: temporal and energy-related features (duration, power) demonstrated a potential for being a discriminant tool between the two forage species, grasses or legumes. Despite these differences, no different level of sound perception was highlighted in the recognition of grazing behavior between the two swards, due to the marked selective activity of ewes towards legumes, even on the grass-rich one. Future developments include the extension of the dataset, including more individuals and forage types, the application of deep learning-based classification models, and real-time automated analysis using embedded devices.

## Figures and Tables

**Table 1:** Biomass, chemical and botanical composition of swards.

	Grass-Rich Sward	Legume-Rich Sward	*p*
Biomass t/Ha	1.64	4.37	0.240
Dry matter (DM) %	31.2	34.4	0.327
Crude protein % DM	11.7	13.3	0.260
NDF % DM	55.4	43.2	0.085
Sugars % DM	16.0	13.3	0.032
Fat % DM	3.21	2.56	0.007

**Table 2:** Botanical composition of swards and of the selected diets and selectivity indexes.

	Grass-Rich Sward	Legumes-Rich Sward	*p*
Botanical composition of swards %			
Grasses	59.2	29.1	0.145
Legumes	3.4	57.5	0.019
Other essences	37.4	13.3	0.120
Botanical composition of the selected diets %			
Grasses %	26.1	17.8	0.067
Legumes %	10.5	59.4	<0.001
Other essences %	61.1	22.7	<0.001
Selectivity index SI			
SI grasses	0.44	0.61	0.153
SI legumes	3.10	1.03	0.006
SI other essences	1.63	1.71	0.812

SI, selectivity index: percentage of selected essence/percentage of available essence (SI < 0.7, rejected; SI 0.7–1.3, neutral; SI 1.3–2.5, preferred; SI > 2.5, highly preferred [20].

**Table 3:** Eating, rumination and other activities time and the number of herbage prehensions. Comparisons between audio and video detections and between swards.

	Detection Method (DM)		Sward (S)		*p*		
	Audio	Video	G	L	DM	S	DM × S
Eating time (minutes)	9.19	8.80	8.81	9.19	0.332	0.359	0.806
Rumination time (minutes)	0.32	0.49	0.51	0.30	0.325	0.244	0.576
Other activities (minutes)	3.84	4.10	3.63	4.31	0.447	0.046	0.674
Prehensions number	334	339	345	328	0.804	0.333	0.952

**Table 4:** Percentages of true positives, false positives and false negatives, precision and recall obtained by the comparison between herbage prehensions estimated with audio recordings and detected with video recordings in the two swards.

	Grass-Rich Sward	Legumes-Rich Sward	*p*
True positives (TP) %	76	75.1	0.539
False positives (FP) %	11.7	12.6	0.703
False negatives (FN) %	11.9	12.3	0.773
Precision %	0.78	0.80	0.404
Recall %	0.78	0.80	0.207

Precision: TP/(TP + FP); Recall: TP/(TP + FN).

**Table 5:** Simple linear regression analysis between sound and video detection for number of prehensions, eating time and rumination time in the two swards. R^2^ and *p* values.

	Grass-Rich Sward		Legume-Rich Sward	
	R^2^	*p*	R^2^	*p*
Number of prehensions	0.743	<0.001	0.740	<0.001
Eating time	0.783	<0.001	0.734	<0.001
Rumination time	0.950	<0.001	0.354	<0.001

**Table 6:** Means values of the main acoustic features extracted from audio segments for grass and legume bites.

	Grass Bites	Legume Bites	*p*
Duration, s	0.29	0.36	<0.001
Pitch, Hz	228.3	186.6	0.001
Power, dbFS ^1^	−29.8	−33.8	<0.001
ZCR ^2^	0.06	0.08	0.001

^1^, decibels relative to full scale; ^2^, zero crossing rate.

## Data Availability

The Agritech National Research Center is setting up a dedicated platform to host a public repository of datasets generated by the research developed within the Agritech spoke 5 project titled “Sustainable productivity and mitigation of environmental impact in livestock systems”. At the date of submission of the manuscript, the repository is not yet active. The data presented in this study are available on request from the corresponding author.
